# Effect of Lower- versus Higher-Intensity Isometric Handgrip Training in Adults with Hypertension: A Randomized Controlled Trial

**DOI:** 10.3390/jcdd9090287

**Published:** 2022-08-30

**Authors:** Mohsen Javidi, Sajad Ahmadizad, Hassan Argani, Abdolrahman Najafi, Khosrow Ebrahim, Narges Salehi, Yasaman Javidi, Linda S. Pescatello, Alireza Jowhari, Daniel A. Hackett

**Affiliations:** 1 Department of Biological Sciences in Sport, Faculty of Sports Sciences and Health, Shahid Beheshti University, Tehran 19839-69411, Iran; 2 Urology and Nephrology Research Center, Beheshti University of Medical Sciences, Tehran 19839-63113, Iran; 3 Department of Physical Education and Sport Sciences, Science and Research Branch, Islamic Azad University, Tehran 19168-93813, Iran; 4 Department of Midwifery, School of Nursing and Midwifery, Shiraz University of Medical Sciences, Shiraz 71348-14336, Iran; 5 Department of Kinesiology, College of Agriculture, Health and Natural Resources, University of Connecticut, Mansfield, CT 06269, USA; 6 Department of Sport Science, Faculty of Education and Psychology, Shiraz University, Shiraz 71348-14336, Iran; 7 School of Health Sciences, Faculty of Medicine and Health, The University of Sydney, Camperdown, NSW 2006, Australia

**Keywords:** blood pressure, resistance training, isometric exercise, cardiovascular health, cardiovascular risk

## Abstract

This study compared the effects of lower- versus higher-intensity isometric handgrip exercise on resting blood pressure (BP) and associated clinical markers in adults with hypertension. Thirty-nine males were randomly assigned to one of three groups, including isometric handgrip at 60% maximal voluntary contraction (IHG-60), isometric handgrip at 30% IHG-30, or a control group (CON) that had been instructed to continue with their current activities of daily living. The volume was equated between the exercise groups, with IHG-60 performing 8 × 30-s contractions and IHG-30 performing 4 × 2-min contractions. Training was performed three times per week for 8 weeks. Resting BP (median [IQR]), flow-mediated dilation, heart rate variability, and serum markers of inflammation and oxidative stress were measured pre- and post-intervention. Systolic BP was significantly reduced for IHG-60 (−15.5 mmHg [−18.75, −7.25]) and IHG-30 (−5.0 mmHg [−7.5, −3.5]) compared to CON (*p* < 0.01), but no differences were observed between both the exercise groups. A greater reduction in diastolic BP was observed for IHG-60 (−5.0 mmHg [−6.0, −4.25] compared to IHG-30 (−2.0 mmHg [−2.5, −2.0], *p* = 0.042), and for both exercise groups compared to CON (*p* < 0.05). Flow-mediated dilation increased for both exercise groups versus CON (*p* < 0.001). IHG-30 had greater reductions in interleukin-6 and tumor necrosis factor-α compared to the other groups (*p* < 0.05) and CON (*p* = 0.018), respectively. There was a reduction in Endothelin-1 for IHG-60 compared to CON (*p* = 0.018). Both the lower- and higher-intensity IHG training appear to be associated with reductions in resting BP and improvements in clinical markers of inflammation and oxidative stress.

## 1. Introduction

Hypertension, or high blood pressure (BP), is a major risk factor for cardiovascular disease and disability [1]. The prevalence of high BP and the estimated associated deaths have substantially increased over the past three decades [2]. Typically, people with high BP also have other cardiovascular disease risk factors, such as cigarette smoking, dyslipidemia, overweight, diabetes, physical inactivity, and an unhealthy diet [3]. Thus, the current guidelines recommend lifestyle modifications (e.g., increased physical activity and stress management) as the first line of treatment for high BP [3].

Exercise is one of the most effective antihypertensive non-pharmacological strategies [4]. Hanssen et al. [5] performed a systematic review of 34 meta-analyses to develop a scientific Consensus Document for individualized exercise prescription for people at risk of developing hypertension and with hypertension. Based on this work, aerobic training was recommended as the first line exercise therapy for people with high BP (≥140 mmHg systolic and/or ≥90 mmHg diastolic). Dynamic resistance training was considered a second-line exercise treatment for people with high BP, due to the smaller BP reductions observed [5]. However, the effectiveness of an exercise intervention for reducing BP appears to be influenced by the individual initial BP. This is supported by the recommendation by Hanssen et al. [5] for dynamic resistance training rather than aerobic training being a first-line exercise priority for people with high-normal BP (130–139 mmHg systolic and/or 85–89 mmHg diastolic). 

Hansen et al. [5] recommended that isometric resistance training be given equal priority to dynamic resistance training for people with high BP, and suggested that it could elicit similar if not superior BP reduction as dynamic resistance training in people with high-normal BP. Additionally, the American College of Cardiology and the American Heart Association (ACC/AHA) [3] have stated that results from previous meta-analyses [6,7,8] suggest that isometric resistance training substantially lowers BP. However, there was no conclusion about the antihypertensive benefits of isometric resistance training in the American College of Sports Medicine (ACSM) Pronouncement on the role of physical activity to prevent and treat hypertension [9]. This was due to a lack of large, well-designed randomized controlled trials among adults with hypertension comparing isometric resistance training to aerobic training. Therefore, Pescatello et al. [10] advises caution when making conclusions about the treatment of hypertension with isometric resistance training.

The most common isometric resistance training prescription involves 4 sets × 2 min of handgrip or leg contractions (1–4 min rest between sets) at 30–50% maximal voluntary contraction (MVC), completed 3–5 times per week [11,12]. However, to date, there is a paucity of research investigating the influence of the intensity (i.e., % MVC) used during isometric resistance training on BP in adults with hypertension. Smart et al. [11] conducted a meta-analysis exploring the effect of isometric resistance training on BP in adults based on individual participant data (sex, antihypertensive medication status, BMI, age, hypertensive status, exercise type, and study design variables). The findings showed that unmedicated compared to medicated participants displayed a trend towards a greater reduction in BP, while the other variables did not influence the effect following this type of exercise. Specific exercise prescription variables such as intensity (e.g., % MVC) were not investigated in the Smart et al. meta-analysis [11].

An intensity-dependent reduction in resting blood pressure has been found in hypertensive and pre-hypertensive males [13], and normotensive males [14,15]. However, another study in normotensive adults comparing lower and higher intensities found no difference in blood pressure reduction effects between groups assigned to training with either 5% or 10% MVC [16]. All these previous studies examining lower and higher intensities on resting BP have not compared intensities of above 30% MVC. We previously showed that an acute bout of isometric handgrip exercise performed at 60% MVC can induce post-exercise hypotension in people with high BP, but no post-exercise hypotension was found when using 30% MVC [17]. Additionally, all participants were able to complete isometric handgrip exercise using 60% MVC. Therefore, it is plausible that the more intensive training stimulus associated with 60% MVC compared to 30% MVC may be more efficacious for promoting reductions in resting BP in people with hypertension.

The mechanism responsible for the reduction in resting BP after isometric resistance training is unclear. It appears that increased endothelial-dependent vasodilation, a marker of nitric oxide bioavailability, could be a main mechanism [18]. Additionally, an improvement in autonomic neural network balance has been shown following isometric resistance training [19], and is associated with the improvement of local endothelial function [20] and a reduction in oxidative stress [21]. If isometric resistance training improves endothelial function, cardiac autonomic modulation, and oxidative stress in adults with hypertension, it is plausible there would also be a reduction in inflammation [22].

The aim of this study was to examine the chronic effect of lower- versus higher-intensity, volume (load × repetition duration) equated, isometric handgrip exercise on resting BP in adults with hypertension. We hypothesize that higher- compared to lower-intensity isometric handgrip exercises would lead to a greater reduction in resting BP. It was also hypothesized that an improvement in endothelial function, cardiac autonomic modulation, oxidative stress, and inflammation would result following isometric handgrip training, compared to a control group.

## 2. Materials and Methods

### 2.1. Participants

**Figure 1:**
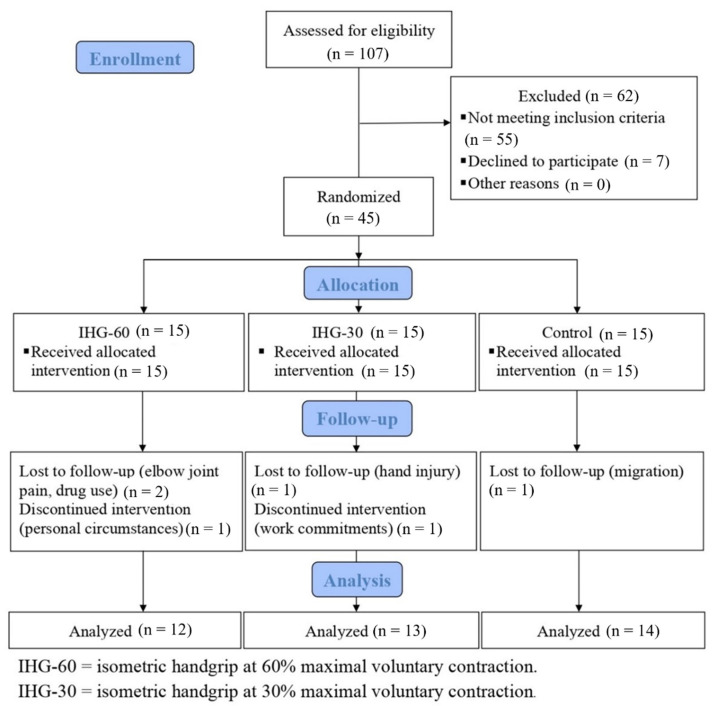
Study flow chart.

Thirty-nine males (age: 46.1 ± 6.4 y; height: 176.9 ± 6.9 cm; weight: 87.0 ± 15.7 kg) were screened by a specialist physician and selected to participate in this study. To be eligible for the study, potential participants needed to have stage 1 or stage 2 hypertension according to the ACC/AHA hypertension guidelines [23]. Additionally, there needed to be an absence of any underlying disease and no history of regular physical activity within the previous six months. Other exclusion criteria included smoking, current medication use (that affected cardiovascular responses, such as blood pressure medication), and physical limitations in performing isometric handgrip exercises. The recruitment of participants was made through local media advertising at Shahid Beheshti University. Participants were randomly assigned to one of three groups, including higher-intensity isometric handgrip at 60% MVC (IHG-60) (*n* = 12), lower-intensity IHG at 30% MVC (IHG-30) (*n* = 13), or a control group (CON) (*n* = 14). The study flow chart is presented in Figure 1. The study was approved by the University Research and Ethics Committee, and all participants voluntarily provided written informed consent (IR.SBU.ICBS.97/1024).

### 2.2. Intervention

Participants randomized to the IHG groups performed three sessions per week for 8 weeks. Participants randomized to CON were instructed to continue with their current activities of daily living, and to attend the clinic once per week for a review of their BP. The IHG-60 and IHG-30 protocols were performed with the non-dominant hand using a digital dynamometer (Saehan Grip, DHD-3 model, made in South Korea). The IHG-60 protocol involved 8 sets of 30 s contractions at 60% maximal voluntary contraction (MVC), with two minutes inter-set recovery. The IHG-30 protocol involved 4 sets of 2 min contractions at 30% MVC with 4 min inter-set recovery. There was a similar duration for the IHG-60 and IHG-30 protocols (18 and 20 min, respectively). The training volume (load × duration of contractions) was matched between the groups (14,400 arbitrary units). An assessment of MVC of the non-dominant hand was conducted at the beginning of each week of the intervention for participants in the IHG groups, to ensure that the targeted training intensity was maintained. Training sessions were performed in the morning between 8:00 to 12:00 in a clinical environment (i.e., a quiet room) under the direct supervision of a researcher. Each week throughout the intervention, all groups were reminded to participate in no other exercise program, not make any changes to their diet, and avoid drugs and all substances influencing BP. For participants in IHG-60 and IHG-30, their recent diet and physical activity were reported and recorded during each training session. The MVC of the non-dominant hand was also assessed pre- and post-intervention for all participants to determine the effectiveness of the exercise interventions on muscle performance adaptations.

### 2.3. Pre- and Post-Intervention Assessments

Pre- and post-intervention assessments were conducted across two days during the morning in fasted states (48 h after the IHG sessions). Participants were reminded to abstain from any caffeinated substances two days before the assessments, and reported a lifestyle in which they abstained from alcohol. The participants were instructed to have a least 6 h of sleep the night before the assessments. The flow-mediated dilation and heart rate variability measures were taken in one session by a specialist who was blinded to group allocation. For this session, participants were also instructed to refrain from any strenuous physical or emotional activity in the prior 24 h. All other measures (anthropometry, BP, serum markers of inflammation and oxidative stress, and MVC) were taken in the other session.

### 2.4. Blood Pressure Measurement

Participants rested (for 15 min) quietly in a comfortable chair with their upper arm at heart level. BP was taken on the dominant arm and at the brachial artery with an automated noninvasive BP monitor (Omron M6 Comfort, HEM-7221-E, Omron Healthcare, Kyoto, Japan) in accordance with recommendations [24]. Since the accuracy of the BP measurements can be influenced by numerous factors, the methodology followed will be briefly described [25,26]. To improve the accuracy of the BP measurements, participants remained silent, with the room also quiet. Participants sat in the chair comfortably, with legs uncrossed, and their back and arm supported. The middle of the cuff was positioned on the upper arm, with the arm elevated to be at the level of right atrium (i.e., the mid-point of the sternum). The lower end of the cuff was positioned 2–3 cm above the antecubital fossa. Participants were instructed to remove all clothing that covered the location where the cuff was placed. BP was measured three times, and the average of the last two measurements was used to represent resting systolic BP (SBP), diastolic BP (DBP), and heart rate (HR). Participants were not aware of the BP reading at the time of measurement. Mean arterial pressure (MAP) was calculated using the following equation: MAP = DBP + 1/3 (SBP-DBP).

### 2.5. Flow-Mediated Dilation Measurement

For this assessment, participants were required to rest in a supine position for 20 min, and an occlusion cuff was placed around the forearm of the non-dominant hand. The flow-mediated dilation (FMD) of the brachial artery was measured and recorded with a Doppler ultrasound device (AtCor Medical, Solingen, Germany). The ultrasound probe (7.5 MHz probe) was placed 3 to 5 cm above the elbow cavity of the non-dominant hand to allow for the measurement of the anterior–posterior diameter of the brachial artery, and this location was marked to ensure image consistency. The occlusion cuff was inflated to 50 mmHg above SBP for 5 min, which occluded blood flow below the arterial scan site, causing ischemia. After the 5 min of forearm ischemia, the cuff was deflated and 3 min later, the anterior–posterior diameter of the brachial artery was measured from the marked location. The largest diameter was recorded for the FMD index at the end diastole. The % FMD was calculated using [(Maximum Diameter − Rest Diameter)/Rest Diameter] × 100.

### 2.6. Heart Rate Variability Measurement

Heart rate variability (HRV) was assessed using a Holter heart monitoring device (DMS-services, VX3+ Holter recorder, Los Angeles, CA, USA). Participants were assessed after lying in a supine position for 15 min in a semi-dark and quiet room at a temperature of 22 to 24 °C. Seven ECG recording electrodes were attached to the chest of participants according to the device manual. The participants’ heart rate (R-R intervals) was recorded for 20 min and then analyzed with software (DMS-services, Holter analysis software, Los Angeles, CA, USA). The time axis parameters calculated included SDNN (standard deviation of R-R) and pNN50 (% of successive R-R intervals that differ by more than 50 ms) [27]. Briefly, an increase in SDNN and pNN50 leads to an increase in HRV, and is usually present with lower HR values. The frequency axis parameters calculated were low frequency-LF (% of bands belonging to 0.04 to 0.15 Hz), reflecting the sympathetic activity of the heart, and high frequency-HF (% of bands belonging to 0.15 to 0.4 Hz) reflecting the parasympathetic activity of the heart [27]. Additionally, the LF/HF ratio was calculated to determine the predominance of the baroreflex activity or the vagal modulation of HR [28]. Briefly, an increase in heart activity is indicated by increases in LF and LF/HF ratios, and may reflect a stressful condition [27].

### 2.7. Serum Markers of Inflammation and Oxidative Stress

The blood samples collected were centrifuged for 10 min at 3500 rpm to separate the serum, and then stored at minus 80 °C. Interleukin-6 (IL-6) and tumor necrosis factor-α (TNF-α) levels were measured using the enzyme-linked immune sorbent assay (ELISA) (Diaclone, Besançon, France). The ELISA method and special kits were used to measure Endothelin-1 (ET-1) (CUSABIO Biotechnology Company, Tokyo, Japan), malondialdehyde (MDA), and total antioxidant capacity (TAC) (ZellBio GmbH, Ulm, Germany). Levels of carbonyl protein (CP) were measured using a chemical colorimetric assay kit (Cayman Chemical Company, Ann Arbor, MI, USA).

### 2.8. Statistical Analysis

Statistical analyses were performed using IBM SPSS Statistics ver. 24.0 (IBM Co., Armonk, NY, USA). Data were statistically assessed for normality using the Shapiro-Wilk test. The normality of data distribution was inconsistent, and given the relatively small sample size, it was decided that non-parametric tests would be used for all analyses. The data were presented as median with interquartile range (IQR). Group differences at the baseline were assessed using Kruskal–Wallis tests. Wilcoxon signed-rank tests were performed to evaluate the intervention effect (within-group) by time from baseline to post-intervention. Kruskal–Wallis tests were used to determine the between-group differences at post-intervention for the three groups on the delta score (post-intervention score minus baseline score). When significant between-group differences were identified, Dunn’s pairwise tests and post hoc Bonferroni correction were performed. The significance level was set at *p* < 0.05 (two-tailed).

## 3. Results

There was >80% compliance with the training sessions, and all participants performed the post-intervention assessments. No adverse events were reported for any of the groups. There was no statistical difference between the groups at baseline for any outcome. A significant within-group increase in handgrip MVC occurred for IHG-60 (11.8%; IQR: 7.3, 19.9; *p* = 0.002) and IHG-30 (13.6%; IQR: 10.0, 18.5; *p* = 0.001). There was a significant between-group difference (X_2_ = 24.67, *p* ≤ 0.001), with post hoc tests showing greater MVC increases for both exercise groups, compared to CON (*p* < 0.001).

### 3.1. Blood Pressure

**Figure 2:**
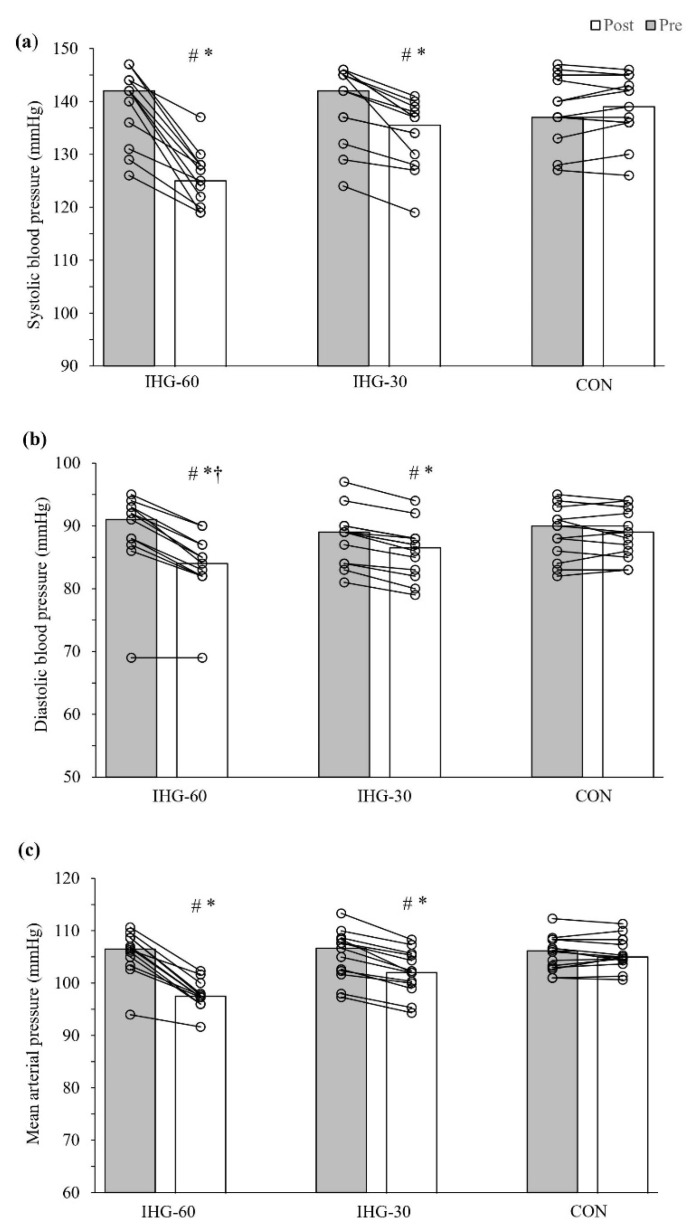
Blood pressure, pre- and post-intervention. Bars represent the pre- and post-intervention median: (**a**) systolic blood pressure, (**b**) diastolic blood pressure, and (**c**) mean arterial pressure for the groups. Paired scatterplots show individual changes for each participant. IHG-60 = isometric handgrip performed at 60% maximal voluntary contraction; IHG-30 = isometric handgrip performed at 30% maximal voluntary contraction; CON = control. # Significant difference compared to pre- (*p* < 0.01); * Significant difference compared to CON (*p* < 0.001); † Significant difference compared to IHG-30.

Significant within-group reductions in SBP were found for IHG-60 (−15.5 mmHg; IQR: −18.75, −7.25; *p* = 0.002) and IHG-30 (−5.0 mmHg; IQR: −7.5, −3.5; *p* = 0.001), with no changes for CON (Figure 2). There were significant between-group differences for SBP (X_2_ = 31.19, *p* ≤ 0.001), with the post hoc test revealing a reduction in SBP for both exercise groups compared to CON (*p* < 0.01). However, there was no difference between IHG-60 and IHG-30 for reductions in SBP (*p* = 0.078). Significant within-group reductions in DBP were found for IHG-60 (−5.0 mmHg; IQR: −6.0, −4.25; *p* = 0.003) and IHG-30 (−2.0 mmHg; IQR: −2.5, −2.0; *p* = 0.001), while no changes occurred for CON (Figure 2). Significant between-group differences for DBP were found (X_2_ = 25.60, *p* ≤ 0.001), with post hoc tests revealing a greater reduction in DBP for IHG-60 compared to IHG-30 (*p* = 0.042), and for both exercise groups compared to CON (*p* < 0.05). Significant within-group reductions in MAP were found for IHG-60 (−9.17 mmHg; IQR: −9.91, −5.42; *p* = 0.002) and IHG-30 (−3.0 mmHg; IQR: −4.0, −2.5; *p* = 0.001), while no changes occurred for CON (Figure 2). There were significant between-group differences for MAP (X_2_ = 30.72, *p* ≤ 0.001), with post hoc tests revealing a greater reduction in MAP for both exercise groups compared to CON (*p* < 0.01). However, there was no difference between IHG-60 and IHG-30 for reductions in MAP (*p* = 0.074).

### 3.2. Flow-Mediated Dilation and Resting Heart Rate

Significant within-group increases for FMD occurred for IHG-60 (*p* = 0.002) and IHG-30 (*p* = 0.001), whereas a significant within-group decrease occurred in CON (*p* = 0.03) (Table 1). There was a significant between-group difference (X_2_ = 27.29, *p* < 0.001), with the post hoc tests revealing greater FMD increases for both exercise groups, compared to CON (*p* < 0.001). The resting HR was significantly decreased pre- to post-intervention for IHG-60 (*p* = 0.002) and IHG-30 (*p* = 0.001), but not in CON (Table 1). A significant between-group difference was found (X_2_ = 21.70, *p* ≤ 0.001), with the post hoc tests showing greater reductions in resting HR for IHG-60 compared to IHG-30 (*p* = 0.046) and CON (*p* < 0.001). 

### 3.3. Heart Rate Variability

For the heart rate variability measures, SDNN and PNN50 increased for all groups (*p* < 0.05) (Table 1). IHG-30 showed a significant pre- to post-intervention decrease in HF (*p* = 0.036), and a significant increase in LF/HF (*p* = 0.007), with no significant changes for these specific measures for other groups. Both IHG-60 and CON showed a significant decrease in LF (*p* = 0.015 and *p* = 0.002, respectively), but no significant change for IHG-30. There were no significant between-group differences for any of the heart rate variability measures.

### 3.4. Inflammation and Oxidative Stress

The results for the serum markers of inflammation and oxidative stress within- and between groups are illustrated in Table 2. A significant decrease in CP from pre- to post-intervention occurred for IHG-60 (*p* = 0.004), but no significant between-group difference. A significant within-group increase in IL-6 occurred for IHG-60 (*p* = 0.031), while IHG-30 showed a significant decrease in IL-6 (*p* = 0.009). There was a significant between-group difference for IL-6 (X_2_ = 13.96, *p* = 0.001), with post hoc tests revealing a significant reduction in IL-6 for IHG-30, compared to IHG-60 (*p* = 0.001) and CON (*p* = 0.032). A significant within-group decrease in TNF-α occurred for IHG-30 (*p* = 0.022), and an increase was found for CON (*p* = 0.035). There was a significant between-group difference for TNF-α (X_2_ = 8.15, *p* = 0.017). The post hoc tests revealed that TNF-α was significantly reduced for IHG-30 compared to CON (*p* = 0.018). A significant within-group decrease in ET-1 was found for IHG-60 (*p* = 0.041), and there was a significant between-group difference (X_2_ = 7.66, *p* = 0.022). The post hoc tests revealed a significant reduction in ET-1 for IHG-60 compared to CON (*p* = 0.018). There were no significant within- or between-group differences for TAC and MDA.

## 4. Discussion

This study aimed to compare lower- versus higher-intensity IHG exercise on resting BP and associated clinical markers in men with hypertension. Both IHG-60 and IHG-30 experienced greater reductions in resting BP compared to CON. In partial agreement with the original hypothesis, there was a greater reduction in diastolic BP for IHG-60 compared to IHG-30. FMD increased for both exercise groups compared to CON, while there were no differences between the groups for heart rate variability. There was evidence of a reduction in inflammation for IHG-30 compared to IHG-60 and CON. In contrast, a reduction in a marker of oxidative stress (ET-1) was found for IHG-60 compared to CON. The training stimulus for both exercise groups appeared to be similar, as indicated by a similar increase in muscle performance (MVC) following the 8-week intervention. The compliance to the intervention was high, and no adverse events were reported during the study. Both lower- and higher-intensity IHG training appear to be associated with reductions in resting BP, and improvements in the clinical markers of inflammation and oxidative stress. These findings build upon the evidence for the effectiveness of isometric resistance training for the prevention and treatment of hypertension, thus reducing the risk for cardiovascular disease and disability. 

Findings from a systematic review and subsequent meta-analysis by Loaiza-Betancur and Chulvi-Medrano [29] confirmed that people with high BP can reduce their resting BP through lower-intensity (≤50% MVC) IHG training. However, it was unknown whether IHG training with a higher intensity (with groups being volume-equated) would elicit greater improvements, commonly referred to as a ‘dose-response’ effect [30]. We found improvements in SBP and MAP for both IHG exercise groups, compared to CON. For these BP parameters, there was a trend towards significance in favor of greater reductions for IHG-60, compared to IHG-30 (SBP: −15.5 mmHg versus −5.0 mmHg) and MAP (−9.2 mmHg versus −3.0 mmHg). Consistent with the other BP results, there was a greater reduction in DBP for the exercise groups compared to CON; however, the reductions in DBP were significantly greater for the higher- compared to lower-intensity IHG group (−5.0 mmHg versus −2.0 mmHg). These BP results suggest that higher-intensity IHG exercise may lead to greater improvements in resting BP. 

There have been four previous studies that have directly compared lower- versus higher-intensity isometric resistance training [13,14,15,16]. Three of these studies found greater reductions in resting BP following training at higher intensities, although the higher intensity used did not exceed 30% MVC. When performing IHG exercises at higher intensities there would be an expectant increase in intramuscular pressure, a decrease in active skeletal muscle blood flow, and increases in anaerobic activity [31]. The substantial difference between the intensities of IHG exercise in the present study (i.e., 60% versus 30% MVC) likely resulted in different hemodynamic demands during the training sessions, between the exercise groups. In contrast, the previous studies that investigated this research topic probably used intensities that were too similar, with an approximate difference of only 10% MVC. It should also be considered that resting BP responses following IHG training have been shown to be influenced by the population examined (e.g., normotensive versus hypertensive) [8], as well as by other factors, such as being medicated [11].

The present study finding of improvement in the brachial artery FMD following IHG training is consistent with previous reports in adults with hypertension [20,32]. A potential cause for the improved FMD after IHG training are shear-stress mediated increases in nitric oxide availability [33]. Additionally, an improvement in oxidative stress may also play a role in improving FMD following IHG training [21]. In our study, reductions in resting BP were concomitant with the improvement in FMD. An improvement in resting BP following isometric exercise is thought to be influenced by a reduction in total peripheral resistance, due to enhanced endothelium-dependent vasodilation [12]. However, the results of the present study do not support the assumption that increases in nitric oxide availability or an improvement in oxidative stress led to the reductions in FMD and BP. It should also be noted that changes in resistance vessel endothelial function do not always accompany reductions in BP after IHG training [34]. Therefore, it is unknown what specific factors led to the improvement in FMD and BP following the IHG interventions. Nonetheless, FMD is an independent risk factor for cardiovascular disease, and improvement in FMD alone is of great benefit to people with high BP [35].

For people with hypertension, increased inflammation and oxidative stress are thought to play a role in the pathophysiology of this condition [36]. Only IHG-30 showed a consistent improvement in their inflammatory state, with reductions in IL-6 and TNF-α compared to CON, and compared to IHG-60 for IL-6. As for oxidative stress, IHG-60 had a reduction in ET-1 when compared to the CON, but there was no evidence of IHG-30 decreasing oxidative stress. Since both IHG groups had a reduction in resting BP, it appears that IHG training improves resting BP independent of changes in inflammation and oxidative stress. Inflammation and oxidative stress are closely related pathophysiological processes, and are implicated in many chronic diseases [37]. Therefore, since reductions in oxidative stress and inflammation were found following the exercise interventions, IHG training may have a positive impact upon the disease risk profiles of men with hypertension. However, the different responses to inflammation and oxidative stress between the exercise groups appears to be influenced by the IHG training protocol. The different acute physiological responses of the two exercise protocols used in the present study has previously been observed with higher blood lactate contraction following the higher- compared to lower-intensity IHG exercise [17]. Further research is needed to examine how to optimize IHG protocols to produce favorable changes in inflammation and oxidative stress.

Although there were no differences between the groups for the heart rate variability measures, there were some interesting within-group findings. At baseline, the median SDNN values for all groups was just above 50 ms, which is considered as having compromised health [38], and there was a slight improvement for all groups post-intervention. A lower risk of mortality has been found among cardiovascular disease patients with SDNN values over 100 ms compared to under 50 ms [38]. However, it is unclear as to whether increasing SDNN following training would reduce the risk of mortality. All groups improved PNN50, which suggests there was an increase in parasympathetic nervous system activity [39]. For IHG-30, there was a decrease in HF and increase in LF/HF, indicating a sympathetic dominance that is observed during times of stress, panic, anxiety, or worry [40]. A decrease in LF was observed in IHG-60 and CON, which provides evidence of reduced sympathetic activity of the heart [27]. Only the exercise groups showed a decrease in resting heart rate, with greater reductions for IHG-60 compared to IHG-30. The greater reduction in resting heart for the higher-intensity IHG group is consistent with changes in parasympathetic and sympathetic nervous system activity observed in the IHG groups. Therefore, it does appear that cardiac autonomic modulation does change following IHG training, with potentially more favorable responses when using a higher intensity.

There are some limitations in the current study that should be noted. Even though participants were instructed not to change their diets or physical activity during the study, it is possible that this may have occurred. However, we attempted to monitor any changes throughout the intervention by asking participants each week about any changes to their diet and physical activity. The sample size was relatively small and may have underpowered the study to detect significant differences between groups (e.g., SBP and MAP). Nonetheless, non-parametric statistical tests, which are more conservative than parametric tests and will less likely lead to a false rejection of the null hypothesis [41], were used to assess all data. The exercise intervention lasted 8 weeks which is similar to the duration of previous studies conducted on this topic. As an example, the studies included in the systematic review with a meta-analysis by Smart et al. [11] had interventions ranging from 4 to 12 weeks. It would be of interest to investigate the effects of 60% versus 30% MVC IHG training in people with hypertension over a longer duration, to examine the BP reduction effects as well as the adherence to utilizing higher intensities. However, it should be emphasized that the current study is novel due to being the first known to examine different intensities of isometric resistance training in adults with hypertension. Additionally, no study has compared the response of IHG training on BP and associated clinical markers using an intensity above the current recommendations [11,12]. Finally, it is unknown whether prescribing 60% MVC IHG is feasible in community-based settings. Therefore, further research is warranted, to examine the practicality of implementing 60% MVC IHG training in the community.

## 5. Conclusions

IHG training appears to be an effective means of reducing the resting BP and improving other cardiovascular risk factors in men with hypertension. Possibly higher- (60% MVC) compared to lower- (30% MVC) intensity IHG training may promote greater improvements in resting BP, at least for resting diastolic BP. However, a confirmation of the efficacy and safety of higher- compared to lower-intensity IHG training protocols in people with hypertension requires further studies with larger sample sizes.

## Figures and Tables

**Table 1:** Changes in cardiovascular outcomes within- and between groups.

	IHG-60 (*n* = 12)			IHG-30 (*n* = 13)			CON (*n* = 14)			Between-Groups
Variable	Pre	Post	Delta Score	Pre	Post	Delta Score	Pre	Post	Delta Score	*p*-Value
FMD (%)	5.65 (3.15, 7.35)	9.18 (5.0, 12.99)	4.05 (1.65, 5.45), *p* = 0.002 **	5.83 (4.46, 7.07)	8.15 (6.52, 9.89)	2.17 (1.69, 2.63), *p* = 0.001 **	6.04 (4.66, 6.83)	5.54 (4.2, 6.6)	−0.34 (−0.69, 0.05), *p* = 0.03 *	X_2_ = 27.29, *p* < 0.001 **
Resting HR (bpm)	71.5 (65.5, 78.0)	67.0 (61.75, 73.75)	−4.0 (−5.0, −4.0), *p* = 0.002 **	74.0 (67.5, 77.5)	72.0 (65.5, 75.5)	−2.0 (−2.5, −1.0), *p* = 0.001 **	76.0 (67.75, 78.5)	75.0 (67.25, 80.25)	1.0 (−1.0, 1.0), *p* = 0.516	X_2_ = 21.70, *p* < 0.001 **
Heart rate variability										
SDNN (ms)	54.0 (39.0, 67.25)	60.50 (44.75, 67.0)	5.0 (2.0, 11.5), *p* = 0.017 **	54.0 (40.5, 77.0)	55.0 (44.0, 78.0)	4.0 (1.5, 6.5), *p* = 0.002 **	53.0 (48.5, 64.5)	57.0 (52.5, 64.75)	4.5 (2.75, 6.25), *p* = 0.002 **	X_2_ = 0.86, *p* = 0.652
PNN50 (%)	16.0 (13.25, 19.50)	18.0 (14.5, 21.75)	2.0 (1.0, 3.0), *p* = 0.005 **	18.0 (14.0, 24.0)	18.0 (16.0, 26.5)	2.0 (0, 3.0), *p* = 0.019 *	17.5 (14.75, 20.25)	20.5 (16.75, 21.5)	2.0 (1.0, 3.0), *p* = 0.001 **	X_2_ = 0.58, *p* = 0.750
HF (nu)	705.50 (403.50, 879.50)	674.0 (442.5, 927.5)	72.0 (−60.0, 120.75), *p* = 0.347	812.0 (355.0, 935.0)	793.0 (407.0, 991.0)	48.0 (−4.5, 76.0), *p* = 0.036 *	678.0 (566.75, 792.0)	715.5 (648.75, 840.0)	37.5 (3.0, 69.75), *p* = 0.109	X_2_ = 1.23, *p* = 0.541
LF (nu)	1378.0 (996.0, 2104.0)	1329.5 (893.0, 2002.5)	−89.5 (−104.75, −11.5), *p* = 0.015 *	1320.0 (983.5, 2022.0)	1312.0 (945.5, 2013.0)	−24.0 (−56.5, −6.5), *p* = 0.087	1267.0 (1066.75, 1717.75)	1260.5 (1028.75, 1648.75)	−46.0 (−74,25, −15.0), *p* = 0.002 **	X_2_ = 3.51, *p* = 0.173
LF/HF	2.4 (1.38, 4.58)	2.0 (1.43, 2.98)	−0.3 (−0.73, 0.13), *p* = 0.084	1.6 (1.1, 4.8)	1.7 (1.0, 4.1)	−0.2 (−0.6, −0.02), *p* = 0.007 **	1.9 (1.45, 4.33)	1.85 (1.5, 3.45)	−0.25 (−0.85, 0.2), *p* = 0.161	X_2_ = 0.16, *p* = 0.922

FMD = flow-mediated dilation; HR = heart rate; IHG-60 = isometric handgrip performed at 60% maximal voluntary contraction; IHG-30 = isometric handgrip performed at 30% maximal voluntary contraction; CON = control. Data presented as median (interquartile range). Significant effects * *p* < 0.05; ** *p* < 0.01.

**Table 2:** Changes in serum markers of inflammation and oxidative stress within- and between groups.

	IHG-60 (*n* = 12)			IHG-30 (*n* = 13)			CON (*n* = 14)			Between-Group
Variable	Pre	Post	Delta Score	Pre	Post	Delta Score	Pre	Post	Delta Score	*p*-Value
TAC (µmol/lit)	614.5 (541.75, 773.25)	598.5 (541.25, 780.5)	6.0 (−23.5, 19.75), *p* = 0.754	583.0 (496.0, 733.5)	611.0 (511.0, 734.5)	1.0 (−19.5, 12.0), *p* = 0.861	519.5 (418.75, 820.0)	532.0 (429.75, 773.5	−8.0 (−23.25, 10.75), *p* = 0.167	X_2_ = 1.27, *p* = 0.531
MDA (µmol/lit)	21.0 (15.0, 26.5)	19.0 (16.0, 25.25)	−1.0 (−2.75, 1.0), *p* = 0.102	21.0 (12.5, 24.5)	17.0 (13.0, 23.5)	−1.0 (−2.0, 1.0), *p* = 0.265	17.5 (12.75, 24.5)	16.5 (13.0, 23.25)	−0.5 (−3.0, 1.25), *p* = 0.143	X_2_ = 0.12, *p* = 0.940
CP (nmol/mg)	2.14 (1.32, 2.32)	2.06 (1.34, 2.24)	−0.08 (−0.12, −0.07), *p* = 0.004 **	2.17 (1.19, 2.34)	2.17 (1.21, 2.28)	−0.03 (−0.09, 0.08), *p* = 0.552	1.72 (1.1, 2.36)	1.63 (1.2, 2.16)	−0.01 (−0.25, 0.08), *p* = 0.315	X_2_ = 3.51, *p* = 0.173
IL-6 (pg/mL)	1.41 (1.31, 1.55)	1.49 (1.35, 1.68)	0.12 (−0.02, 0.16), *p* = 0.031 *	1.53 (1.45, 1.72)	1.36 (1.22, 1.55)	−0.22 (−0.27, −0.01), *p* = 0.009 **	1.34 (1.22, 1.48)	1.44 (1.26, 1.58)	0.07 (−0.09, 0.11), *p* = 0.431	X_2_ = 13.96, *p* = 0.001 **
TNF-α (pg/mL)	4.65 (3.98, 5.68)	5.2 (4.23, 5.55	0.65 (−0.3, 1.05), *p* = 0.124	4.9 (3.5, 6.15)	4.7 (3.4, 5.75)	−0.3 (−0.55, 0.05), *p* = 0.022 *	4.6 (3.65, 8.18)	6.25 (4.81, 8.31)	0.9 (−0.24, 1.53), *p* = 0.035 *	X_2_ = 8.15, *p* = 0.017 *
ET-1 (pg/mL)	1.12 (0.9, 2.08)	1.08 (0.97, 1.84)	−0.09 (−0.16, 0.01), *p* = 0.041 *	1.07 (0.96, 1.54)	1.12 (0.98, 1.28)	−0.01 (−0.07, 0.07), *p* = 0.674	1.22 (1.02, 1.44)	1.41 (1.15, 1.66)	0.1 (−0.05, 0.35), *p* = 0.059	X_2_ = 7.66, *p* = 0.022 *

TAC = total antioxidant capacity; MDA = malondialdehyde; CP = carbonyl protein; IL-6 = Interleukin-6; TNF = tumor necrosis factor-α; ET-1 = Endothelin-1; IHG-60 = isometric handgrip performed at 60% maximal voluntary contraction; IHG-30 = isometric handgrip performed at 30% maximal voluntary contraction; CON = control. Significant effects * *p* < 0.05; ** *p* < 0.01.

## Data Availability

The data analyzed during this study can be found on the Open Science Framework website: https://osf.io/wv7uz/ (accessed on 20 July 2022).
